# Relevance of prehypertension as a diagnostic category in asymptomatic adults

**DOI:** 10.1590/S1679-45082013000300008

**Published:** 2013

**Authors:** Fernando Costa Nary, Raul D. Santos, Antonio Gabriele Laurinavicius, Raquel Dilguerian de Oliveira Conceição, José Antonio Maluf de Carvalho

**Affiliations:** 1 Hospital Israelita Albert Einstein, São Paulo, SP, Brazil

**Keywords:** Prehypertension, Risk factors, Cardiovascular diseases, Inflammation, C-reactive protein, Metabolic syndrome x

## Abstract

**Objective:**

To assess the association of prehypertension with metabolic, inflammatory and cardiovascular risk profile in asymptomatic individuals.

**Methods:**

Between 2006 and 2009, 11,011 asymptomatic adults (mean age: 43 years; 22% females), underwent a check-up protocol. They were divided into 3 groups: normotensive group (arterial pressure=120/80mmHg), prehypertensive group (arterial pressure >120/80mmHg and <140/90mmHg) and hypertensive group (arterial pressure≥140/90mmHg or prior diagnosis of hypertension). Each group metabolic and cardiovascular group profile was assessed.

**Results:**

The prevalence of normotension, prehypertension and hypertension was 27.9%, 53.9% and 18.2%, respectively. Prehypertensive individuals were older (mean age: 42.7 *versus* 40 years; p<0.001) than normotensive patients, and had higher body mass index (mean: 26.7kg/m^2^ *versus* 24kg/m^2^; p<0.001), higher plasma triglycerides levels (mean: 139mg/dL *versus* 108mg/dL; p<0.001), higher LDL-choleterol levels (mean: 128mg/dL *versus* 117mg/dL; p<0.001), and lower HDL-cholesterol (mean: 46.7mg/dL *versus* 52.7mg/dL; p<0.001). Prehypertensive individuals were more likely to have impaired fasting glucose (OR: 1.69; 95%CI: 1.39-2.04), overweight and obesity - body mass index >25kg/m^2^ (OR: 2.48; 95%CI: 2.24-2.74), hepatic steatosis: (OR: 2.23; 95%CI: 1.97-2.53), metabolic syndrome (OR: 3.05; 95%CI: 2.67-3.49), and high-sensitivity C-reactive protein levels>2mg/L (OR: 1.52; 95%CI: 1.35-1.71).

**Conclusion:**

Prehypertension is associated with an increased prevalence of metabolic syndrome, hepatic steatosis and subclinical inflammation.

## RESUMO

**Objetivo:**

Avaliar a associação da pré-hipertensão com perfis metabólico, inflamatório e de risco cardiovascular em indivíduos assintomáticos.

**Métodos:**

Entre 2006 a 2009, 11.011 adultos assintomáticos (média de idade de 43 anos; 22% mulheres) foram submetidos a protocolo de *check-up,* sendo classificados em 3 grupos: normotensos (pressão arterial<120×80mmHg), pré-hipertensos (pressão arterial= 120/80mmHg e <140/90mmHg) e hipertensos (pressão arterial ≥140/90mmHg ou diagnóstico prévio de hipertensão arterial). Foram avaliados os perfis metabólico e de risco cardiovascular de cada um dos três grupos.

**Resultados:**

A prevalência de normotensão, pré-hipertensão e hipertensão foi, respectivamente, de 27,9%, 53,9% e 18,2%. Quando comparados com os indivíduos normotensos, os pré-hipertensos apresentaram média de idade maior (42,7 *versus* 40 anos; p<0,001), maior índice de massa corporal (média: 26,7kg/m^2^ *versus* 24 kg/m^2^; p<0,001), níveis de triglicérides mais elevados (média: 139mg/dL *versus* 108mg/dL; p<0,001), níveis de LDL-colesterol mais elevados (média: 128mg/dL *versus* 117mg/dL; p<0,001) e níveis séricos menores de HDL-colesterol (média: 46,7mg/dL *versus* 52,7mg/dL; p<0,001). Os pré-hipertensos apresentaram maior prevalência de alterações na glicemia de jejum (OR: 1,69; IC95%: 1,39-2,04), sobrepeso e obesidade - ou seja, índice de massa corporal >25kg/m^2^ (OR: 2,48; IC95%: 2,24-2,74), esteatose hepática (OR: 2,23; IC95%: 1,97-2,53), síndrome metabólica (OR: 3,05; IC95%: 2,67-3,49) e níveis >2mg/L de proteína C-reativa de alta sensibilidade (OR: 1,52; IC95%: 1,35-1,71).

**Conclusão:**

A pré-hipertensão está associada a aumento da prevalência de síndrome metabólica, esteatose hepática e inflamação subclínica.

## INTRODUCTION

Systemic arterial hypertension (SAH) constitutes one of the main modifiable cardiovascular risk factors and also an important public health issue. Death related to cardiovascular disease increases progressively with high blood pressure (BP) from 115/75mmHg in linear form, continuous and independently^(1,2)^. In 2001, about 7.6 million deaths worldwide were attributed to high BP, being the majority of them in low and medium income countries affecting more than half of the population aged between 45 and 69 years old^(3,4)^.

Prehypertension corresponds to BP values ≥120/80mmHg and <140/90mmHg. It was originally introduced in 2003, after publication of the VII Joint National Committee on Prevention, Detection, Evaluation and Treatment of High Blood Pressure (JNC) which aimed to identify a category of individuals within the same BP range, and as a result with higher risk to present SAH in the future^(4)^. The publication also showed that normotensive and prehypertensive individuals had twice more chance to progress to SAH^(5)^ and at the same time a significantly higher cardiovascular risk^(6)^.

However, prehypertension was not globally accepted as a diagnostic category, and it was not adopted in the VI Brazilian Guidelines on SAH^(2)^. Criticisms of nomenclature point out that prehypertension diagnosis identifies a heterogeneous group of individuals who are predominantly healthy with different risk levels to cardiovascular events and to evolve into SAH^(7)^. Most of these healthy individuals could be stigmatized as sick and treated with unnecessary pharmacologic measures^(8)^. Despite that, a prehypertension diagnosis can be a valuable tool to track and sensitize people in order to adopt a healthy life style. Prehypertension is a convergence point for several cardiovascular risk factors, a fact that reinforces the importance of adopting the nomenclature in daily clinical practice aiming to facilitate the encouragement of individuals to adopt a better life style.

## OBJECTIVE

To characterize the metabolic profile and cardiovascular risk factors in a prehypertension population of asymptomatic adults from Brazil, and also to assess the association with conventional risk factors, metabolic syndrome, hepatic steatosis, and BP hyperactivity response to physical exercise and to subclinical inflammation.

## METHODS

We retrospectively evaluated 11,011 Brazilian adults (mean age 43 years old; 22% were women) who underwent a protocol of periodic health evaluation at *Hospital Israelita Albert Einstein* from 2006 to 2009. The protocol was part of the health program for private organizations in the city of São Paulo, which was mandatory and financed by employers. The protocol included several extensive clinical and laboratory evaluations, such as abdominal ultrasonography and treadmill exercise test. Each patient systolic (SBP) and diastolic BP (DBP) was measured following the American Heart Association guidelines^(9)^, using the aneroid sphygmomanometer appropriately measured and calibrated with the cuff fit to the circumference of the arm. The BP was measured three times during evaluation using a mean value. Participants were classified in three groups, according to the BP measurement: normotensive (<120/80mmHg), prehypertensive (≥120/80mmHg and <140/90mmHg) and hypertensive (≥140/90mmHg or earlier diagnosis or SAH).

This study was approved by the Ethical Committee of the *Hospital Israelita Albert Einstein* (CAAE nº 0094.0.028.000-09), and the consent form was dismissed.

### Clinical variables

Individuals with diabetics and earlier diagnosis of cardiovascular disease were excluded. Participants were questioned in relation to previous presence of dyslipidemia, SAH (previous diagnosis of SAH, use of anti-hypertensive medicines or measured BP ≥140/90mmHg), diabetes (previous use of medicines for diabetes or fasting glycemia >126mg/dL) and smoking (consumption of at least one cigarette within the last 30 days). Body mass index (BMI) was measured using the weight/height^2^ (kg/m^2^) formula to classify eutrophic individuals (IMC<25) with overweight (25-29.9) and obese (≥30). Abdominal circumference was evaluated by visceral adiposity. We calculated physical activity level (sendentarism, low physical activity, moderate physical activity and high physical activity according to the IPAQ -International Physical Activity Questionnaire^(10)^.

To get a better evaluation, the sedentary and low activity individuals were grouped in low physical activity group and high physical activity group. Alcohol consumption was measured using the Alcohol Use Disorders Identification Test (AUDIT)^(11)^, being considered moderate/high for score ≥8.

### Laboratorial variables

Clinical biochemical exams were conducted after at least a 12 hour fasting. Total cholesterol, HDL-cholesterol (HDL-c), triglyceride (TG) and glycemia were detected by an enzymatic method on Vitros™ platform (Ortho-Clinical Diagnostic, Inc., Johnson & Johnson, New York, USA). LDL-cholesterol (LDL-c) was calculated using the Friedewald formula for TG<400mg/dL. We considered as altered fasting glycemia values between 100mg/dL and 126mg/dL. Values of high sensitive c-reactive protein (hs-CRP) were determined by the immunoturbidimetry (Dade-Boehring, USA). The presence of subclinic inflammation was considered for hs-CRP values >2,0mg/L.

### Treadmill exercise test to investigate blood pressure with hyper-reactive response during exercise

Treadmill exercise test was conducted using a treadmill and based on Ellestad protocol. The test was interrupted by physical fatigue. We considered BP hyper-reactive response values of SAH >220mmHg and/or elevation of 15mmHg for DBP or higher, considering the normal values for resting BP.

### Abdominal ultrasonography to investigate hepatic steatosis

Patients underwent an abdominal ultrasonography after a minimal of a 6 hours fasting. Hepatic steatosis was defined by ultrasonography standard of the bright liver with evidence of contrast between hepatic and renal parenchyma using the previously described method^(12)^. All exams were conducted using an ACUSON XP-10 (Siemens Company, USA).

### Evaluation of presence of metabolic syndrome and estimated cardiovascular risk

Metabolic syndrome was diagnosed based on the International Diabetes Federation (IDF)^(13)^ criteria. Risk of cardiac death and myocardial infarction in 10 years was calculated using the Framingham score according to gender^(14)^. Participants were grouped as low (<10%) or medium/high risk (≥10%).

### Statistical analysis

All variables were analyzed and statistically compared between prehypertensive and normotensive group. Qualitative measurements were described using relative and absolute frequencies according to groups (normotensive and prehypertensive). The existence of association of these measurements between groups was seen using the χ^2^ test. Quantitative measures were described based on a classification using mean±standard deviation save for variables with abnormal distribution described as median (minimal, maximum). For comparisons the two-sample t test was used and for non-parametric variables, the Mann-Whitney test. The prehypertensive association to cardiovascular risk factor and metabolic parameters was conducted using multivariate analysis initially adjusted for gender and age using general linear models. Subsequently, the evaluated variables were grouped and tested in a multinomial multiple regression model. We calculated odds ratios (OR) with 95% confidence interval for each variable model, taking as reference the prehypertensive group. To verify the continuous relationship between BP values and hs-CRP, dispersive diagrams adjusted to general linear models with gamma distribution and identity link function were created. Tests were conducted considering a significance level of 5%.

## RESULTS

**Figure 1:**
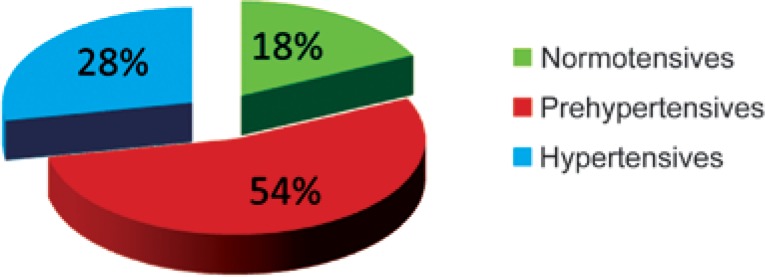
Prevalence of prehypertension

**Figure 2:**
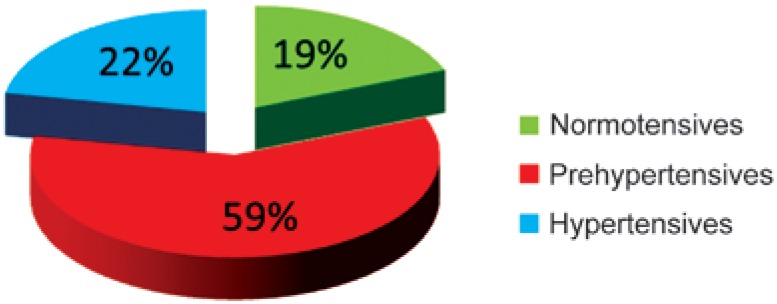
Prevalence of prehypertension in men

**Figure 3:**
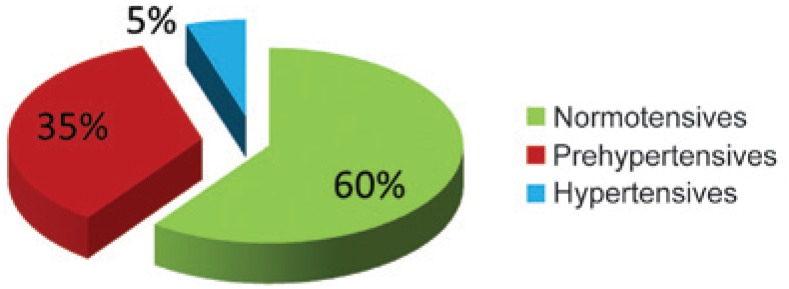
Prevalence of prehypertension in women

Normotension, prehyperthension and hypertension prevalence in the studied group is shown in figure 1. The majority of the population (54%; n=5,946) had prehypertension. Figures 2 and 3 show the prevalence according to gender, being prehypertension more frequent in men.

The comparison of the several clinical and laboratorial variables among normotensive and prehypertensive individuals is shown on table 1. When compared normotensive and prehypertensive individuals, this latter had high BMI, high values of abdominal circumference, and as a result, high prevalence of overweight and obesity.

**Table 1:** Demographic, clinical, hemodynamic and laboratorial variables among normotensive and prehypertensive groups

Variables	Prehypertensive (n=5,932) Mean values (SD)	Normotesive (n=3,076) Medium values (SD)	p value
Age (years)	42.7 (7.3)	40 (6.54)	<0.001
Gender (% women)	14	47	<0.001
BMI (kg/m^2^)	27 (3.7)	24 (3.2)	<0.001
Total cholesterol (mg/dL)	201 (37.8)	191 (35.6)	<0.001
Triglycerides* (mg/dL)	92 (20-840)	118 (18-3,133)	<0.001
LDL-cholesterol (mg/dL)	128 (33.9)	117 (32.8)	<0.001
HDL-cholesterol (mg/dL)	47 (12)	53 (14)	<0.001
Gamma-GT* (U/L)	30 (7-379)	22 (5-375)	<0.001
Fasting glycemia (mg/dL)	91 (15.3)	86 (1.9)	<0.001
hs-CRP* (mg/L)	1 (0.1-10)	1.2 (0.1-10)	<0.001
Smoking (%)	11.6	10.3	0.122
Sedentarism (IPAQ) (%)	25.5	23.8	<0.001
Alcohol consumption AUDIT>8 (%)	14.2	9.1	<0.001
Framingham score >10%	8	3	<0.001

* Medians

SD: standard deviation; BMI: body mass index; hs-CRP: high sensitivity C-reactive protein IPAQ: International Physical Activity Questionnaire; AUDIT: Alcohol Use Disorders Identification Test.

No difference in smoking prevalence between groups was seen (10.3% to normotensives and 11.6% to prehypertensives; p=0.122). There were less individuals practicing physical activity the in prehypertensive group compared with the normotensive group (60.6% and 59.4%, respectively; p<0,001). In addition, a high prevalence of alcohol consumption from moderate to high (14.2% *versus* 9.1% p<0.001) was observed in the prehypertensive group

Table 1 also shows that prehypertensives had high LDL-c values and lower HDL-c values (p<0.001). As a consequence, clinical and laboratorial findings for the prevalence of metabolic syndrome (32% *versus* 11%; p<0.001) and for number of individuals under medium/high risk for coronary disease (8% *versus* 3%; p<0.001) were higher in prehypertensives. Prehypertensive individuals had high prevalence of hepatic steatosis and hyper-reactive response to BP in the treadmill exercise test (p<0.001 for both variables). Medians for hs-CRP were higher in prehypertensives (p<0.001) and, as a consequence, there was a higher prevalence with subclinical inflammation in this group (29% *versus* 23%; p<0.001).

Odds ratio to metabolic, hemodynamic and inflammatory changes between prehypertensives and normotensives after adjustment for age and gender are shown on table 2.

**Table 2:** Prevalence of metabolic syndrome altered fasting glycemia, hepatic steatosis, subclinical inflammation and hyper-reactive response to blood pressure in treadmill test with odds ratio adjusted for age and gender

Variables	Prehypertensives (%)	Normotensives (%)	p values	OR (95%CI)
Metabolic syndrome	31.6	11.1	<0.001	3.05 (2.67-3.49)
Impaired fasting glycemia	11.8	4.8	<0.001	1.69 (1.39-2.04)
Hepatic steatosis	35.9	13.8	<0.001	2.23 (1.97-2.53)
Subclinical inflammation (hs-CRP>2mg/L)	29.2	23.1	<0.001	1.52 (1.35-1.71)
BP hyperreactivity to exercise	9.8	1.6	<0.001	5.06 (3.68-6.95)

95% CI: 95% confidence interval; hs-CRP: high sensitivity C-reactive protein; BP: blood pressure; OR: odds ratio.

Parameters in the adjusted model for all confusion factors are shown on table 3. After adjusts the prehypertensives remained with higher values of LDL-c, higher prevalence of low HDL-c, altered fasting glycemia, metabolic syndrome, altered BP in treadmill exercise test, hepatic steatosis and subclinical inflammation (p<0.05). In fact, after age adjustment there was no difference between groups in prevalence of individuals with medium/high risk for Framingham score. Finally, we observed an increase of 0.018mg/L and 0.025mg/L in hs-CRP levels for each increase of 1mmHg, respectively, in SBP and DBP(p<0.001), after adjustment for confusion factors.

**Table 3:** Odds ratio for metabolic, inflammatory and hemodynamic changes between normotensives and prehypertensives in a multivariate model

Variables	OR	95%CI	p value
BMI>25	1.66	1.46-1.89	<0.001
hs-CRP>2mg/L	1.21	1.06-1.38	0.006
FRS medium/high (>10%)	0.94	0.7-1,26	0.662
Metabolic syndrome	2.14		<0.001
Hepatic steatosis	1.35	1.16-1,58	<0.001
BP HR	4.05	2.87-5.71	<0.001

95%CI: 95% confidence interval; BMI: body mass index; hs-CRP: high sensitive C-reactive protein; FRS: Framingham Risk Score; BP HR: blood pressure hyperactivity response during treadmill exercise test; OR: odds ratio.

## DISCUSSION

This study composed by an opportunist population of asymptomatic individuals submitted to periodic health evaluation, showed that prehypertension was associated in an independent manner after adjustment of confusion factors, with an unfavorable metabolic profile, high prevalence of BP with hyper-reactive response during the treadmill test, hepatic steatosis and subclinical inflammation. In our sample, prehypertension affected more than half of the study population and was significantly more frequent in men, sedentary and overweighed individuals.

Cassani et al. and Silva et al. found prehypertension prevalence of 45% and 36%, respectively, using a sample 10 times lower than the one evaluated in our study^(15,16)^. Similarly, prehypertension in these studies was associated with men, who were overweight and had low degree of physical activity.

Prehypertension is considered the precursor of SAH^(5,8)^. Observation on BP behavior during 50 years in 5,181 participants of the Framingham study revealed that individuals classified as pre-hypertensive evolve to hypertensive stage with higher frequency than normotensive individuals^(5)^. A prospective study comprising prehypertensive individuals that included in its design the development of SAH as one major outcomes showed after that 4 years follow-up, roughly two thirds of prehypertensives (63.0%) evolve to hypertensive status^(8)^. The findings of this study pointed out prehypertension as an independent factor of hyper-reactive response of BP to the exercise.

BP hyper-reactive response during the treadmill test is associated with the appearance of SAH in prospective studies^(17,18)^. Possible explanations to such findings would be the presence of changes in endothelial function and the increased vascular stiffness^(19)^. The independent association observed in this study between prehypertensive and BP hyper-reactive response to exercise reinforces the prehypertension role as a risk factor for posterior development of SAH.

The analyses of several epidemiologic studies revealed that prehypertension is associated with increased risk to develop cardiovascular diseases. A prospective analysis of a cohort study with 8,960 middle aged adults included in the Atherosclerosis Risk in Communities (ARIC) Study showed that the relative risk for cardiovascular disease incidence was 133% higher in individuals with normal to high BP when compared with those with excellent BP (SAH<120mmHg and DBP<80mmHg)^(20)^. In our study prehypertesion was associated with dyslipidemia, dysglycemia and subclinical inflammation regardless of sedentarism, gender, age and overweigh. These findings suggest that prehypertesion could be associated with cardiovascular risk additional to the one caused directly for isolated increase in BP.

Currently, aterosclerosis is considered a proinflammatory status and the hs-CRP high values represent independent markers of cardiovascular risks^(21)^. In our sample those with prehypertesion had 21% more chance to present subclinical inflammation than normotensives. An important factor was the independent association previously described between BP and inflammation^(22)^. For this reason, it is meaningful to report that both BP and inflammation are independent predictors of cardiovascular risks. Besides the fact that the increasing in BP constitutes a proinflammatory status, a possible explanation for an increased hs-CRP in prehypertensives was linked to a high prevalence of hepatic steatosis, even after adjustments on body weight and alcohol consumption. Previous studies showed that non-alcoholic hepatic disease that include hepatic steatosis is associated in an independent manner with an increase in hs-CRP^(23)^. Another physiologic mechanism that could be associated with the findings in our study is the high prevalence of insulin resistance in prehypertensive population. Despite not having measured homeostasis models (HOMA), the high prevalence of dysglycemia in prehypertensive individuals in our study is in agreement with this hypothesis. Indeed, previous studies associated insulin resistance to increased BP, inflammation and hepatic steatosis^(24)^. Toprak et al.^(25)^ also found a positive association between prehypertension and waist circumference, fasting glycemia and plasmatic insulin levels.

Our study findings raise concerns for the high prevalence of prehypertension and other associated factors found in individuals relatively young, which was also reported in other Brazilian studies^(15,16)^. For this reason, it is strongly recommended changes in people's life style and in some cases the use of pharmacological treatment^(26)^ for prehypertensive individuals.

This study cross-sectional design presented a limitation because it was not enable to show causality, but only associations. Therefore, it is not possible to exclude the biased selection related to the population socioeconomic status, whom accessed a systematized periodic health evaluation, a fact that could not be extrapolated to the Brazilian general population. Our results further support previous published Brazilian studies^(14,15)^, with the advantage of analyzing at least 10 times more individuals, which constitutes a significant amount for the parameters studied. Although, after adjustments, we did not see a higher prevalence of individuals with moderate/high cardiovascular risk in the prehypertensive group, it is important to consider the limitations of Framingham score to stratify risks in younger individuals, as depending on age, and often underestimate the cardiovascular risk in this population^(27)^.

## CONCLUSION

Prehypertension is a high prevalent clinical condition associated with independent forms of metabolic syndrome, hepatic steatosis, BP hyper-reactive response during exercise and high degree of subclinical inflammation. To adopt formally prehypertension as a diagnosis category would enable to track and sensitize a significant group of individuals with high cardiovascular risk.
